# *Pentaneurellakatterjokki* Fittkau & Murray (Chironomidae, Tanypodinae): redescription and phylogenetic position

**DOI:** 10.3897/zookeys.833.30936

**Published:** 2019-04-01

**Authors:** Fabio Laurindo da Silva, Elisabeth Stur

**Affiliations:** 1 Department of Natural History, NTNU University Museum, Norwegian University of Science and Technology, NO-7491 Trondheim, Norway Norwegian University of Science and Technology Trondheim Norway; 2 Department of Zoology, Institute of Biosciences, University of São Paulo, CEP: 05508-090, São Paulo, Brazil University of São Paulo São Paulo Brazil

**Keywords:** DNA barcodes, immature stages, non-biting midges, Palearctic, Pentaneurini, taxonomy

## Abstract

The monotypic genus *Pentaneurella* Fittkau & Murray was originally described based on larvae, pupal exuviae and pharate males. The latter prevented the observation of key features, such as wing dimensions, abdominal coloration pattern, and hypopygial apodemes (sternapodeme and phallapodeme), and the description of the adult male was considered incomplete by the authors. Herein, the adult female of *Pentaneurellakatterjokki* is described for the first time, and the adult male, pupa and larva are redescribed and figured based on specimens recently collected in Germany and Norway. We also discuss the phylogenetic position of *Pentaneurella*.

## Introduction

Fittkau (1962), in his reclassification of the subfamily Tanypodinae (Diptera: Chironomidae), separated the genus *Pentaneura* Philippi (*sensu lato*Edwards 1929, Freeman 1955, 1956) into eighteen genera within the tribe Pentaneurini. The only non-South American species assigned to *Pentaneura* sensu Fittkau (1962) was a specimen from northern Sweden, *Pentaneura* spec. Katterjokk (Fittkau 1962), which was later placed in its own monotypic genus, and named *Pentaneurellakatterjokki* (Fittkau & Murray, 1983).

Non-biting midges of the genus *Pentaneurella* are medium-sized dipterans with a Palearctic distribution. Larvae are only known from springs and spring-fed streams in Swedish Lapland and from a mountain stream in northern Norway (Cranston and Epler 2013), although the genus has been consistently recorded through Europe (Sæther and Spies 2013). Fittkau and Murray (1983) described *Pentaneurella* based on larvae, pupal exuviae and pharate males. The latter prevented the observation of key features, such as wing dimensions, wing venation, abdominal coloration pattern, and hypopygial apodemes (sternapodeme and phallapodeme), which are considered essential for male distinction in the subfamily Tanypodinae. Therefore, *Pentaneurellakatterjokki* is redescribed and figured below as adult male, pupa and larva based on specimens recently collected in Germany and Norway. In addition, the adult female is described for the first time. Finally, the phylogenetic position of *Pentaneurella* is discussed.

## Material and methods

Fourth instar larvae and pupae were sampled with hand nets, while adults were collected using emergence- and Malaise traps. Associations between different life stages were established using DNA barcoding. Alcohol-preserved specimens were dissected, the bodies cleared in 8% KOH, and slide-mounted in Euparal®. Measurement methods are according to Epler (1988). Morphological terminology and abbreviations follow Roback (1971) and Sæther (1980), supplemented by Kowalyk (1985) for larval cephalic setation and Silva et al. (2011, 2014) for larval terminology. The color is described based on the specimens preserved in alcohol. The examined specimens are deposited in the NTNU University Museum insect collection (NTNU-VM) and Zoologische Staatssammlung München (ZSM), Germany. One leg was dissected off each specimen and submitted to the Canadian Centre for DNA Barcoding. Metadata, photos, sequences and trace-files are available in the Barcode of Life Data Systems (BOLD, www.boldsystems.org) through the dataset DS-PKATTER with doi: 10.5883/DS-PKATTER. GenBank accessions are HM421431, HM421434, HM421436, HM421438, HM421441 and MK402317 to MK402322. DNA extracts and partial COI gene sequences were generated using standard primers and bi-directional Sanger sequencing with BigDye 3.1 termination at the Canadian Centre for DNA Barcoding in Guelph. Protocols and original trace-files are available through the dataset DS-PKATTER in BOLD. Alignments were done on amino acid sequences and were trivial as indels were absent; only sequences > 300bp were used in the final alignment.

## Taxonomy

### Pentaneurella katterjokki

Taxon classificationAnimaliaDipteraChironomidae

Fittkau & Murray, 1983

Pentaneura spec. Katterjokk Fittkau, 1962: 372 (description of male)
Pentaneurella katterjokki Fittkau & Murray, 1983: 62 (description of male and immature stages)

#### Material examined.

***Type material***: Holotype pharate male (ZSM slide A and B), SWEDEN, Katterjokk, Swedish Lappland, leg. L. Brundin. Two paratypes: pharate female and larva data as for holotype.

***Additional material***: NORWAY, Oppland, Rondane National Park: Adult male (NTNU-VM slide 201765), Skranglehaugen (P4), 1110 m asl, 61.98270N, 9.80360E, 14–21.vii.2008, leg. T. Ekrem, [BOLD ID: ATNA328]. Adult male (NTNU-VM slide 201767), as previous except for Skranglehaugen (P3), 1115 m asl, 61.98219N, 9.80451E, [BOLD ID: ATNA333]. Adult female (NTNU-VM slide 201768), as previous except for 07–14.vii.2008, leg. T. Hoffstad, [BOLD ID: ATNA335]. Adult female (NTNU-VM slide 201766), Skranglehaugen (P2), 1119 m asl, 61.98141N, 9.80480E, 14–21.vii.2008, leg. T. Ekrem, [BOLD ID: ATNA 331]. Pupa (NTNU-VM slide 201769), Skranglehaugen (P5), 1105 m asl, 61.98346N, 9.80384E, 07–14.vii.2008, leg. T. Hoffstad, [BOLD ID: ATNA338]. Larva (NTNU-VM slide 201764) Skranglehaugen, 1117 m asl, benthos, 61.99186N, 9.80454E, 23.vi.2008, leg. E. Stur, [BOLD ID: ATNA122]. Pupa (NTNU-VM slide 201771) Dørålseter, 1032 m asl, kick sample 3, 61.99347N, 9.80343E, 10.viii.2015, leg. K. Hårsaker, T. Ekrem and M. Majaneva, [BOLD ID: EBAI-Ch122]. Larva (NTNU-VM slide 201770) as previous, [BOLD ID: EBAI-Ch66]. GERMANY, Bayern, Berchtesgaden National Park: Adult male, (NTNU-VM slide 201774), Herrenrointquelle 308, 1250 m asl, 47.57778N, 12.97222E, 26.vii-09.viii.2005, leg. F. Eder, [BOLD ID: ES147]. Adult male, (NTNU-VM slide 201772), Schapbachquelle 360a, 1140 m asl, 47.58278N, 12.95806E, 27.v.-14.vi.2005, leg. F. Eder, [BOLD ID: ES46]. Adult male, (NTNU-VM slide 201773), as previous except for 28.vi-12.vii.2005, leg. F. Eder & A. Schellmoser [BOLD ID: ES82].

#### Diagnostic characters.

*Pentaneurellakatterjokki* differs from other Pentaneurini species by the combination of the following characters. ***Adult male***: thorax with a scutal tubercle, tibial spur on fore leg with long outer tooth and shorter side teeth, anal point apically notched. ***Adult female***: gonapophysis VIII triangular, tergite IX without setae, coxosternapodeme strongly curved, postgenital plate broadly rounded, labia with inconspicuous microtrichia. ***Pupa***: plastron plate moderately large, corona absent, anal macrosetae with adhesive sheaths, genital sac symmetrically tapered. ***Larva***: dorsally DP absent, peg sensilla large, firmly fused with the margin of antennal segment 2, forming a fork-like process.

#### Description.

***Adult male* (n = 3, except where otherwise stated).***Size*. Total length 5.2 (1) mm. Wing length 3.0–3.1 mm. Total length/wing length 1.75 (1). Wing length/profemur length 2.09–2.22 (2).

*General coloration*. Head pale brown with darker occipital margin; pedicel and antenna brown; maxillary palp pale brown. Thorax pale brown. Wing membrane transparent without marks. Legs brown to pale brown. Abdominal tergite I–VI white, T VII with continuous pale brown transverse band near proximal margin, VIII pale brown; hypopygium pale brown.

*Head*. Temporal setae 17–19, uniserial. Eye ratio 0.47–0.59. Tentorium 235–289 μm long. Clypeus 132–189 μm long, 97–111 μm wide at widest part, bearing 12–22 setae. Cibarial pump 284–301 μm long. Lengths of palpomeres 1–5 (in μm): 77–82; 84–97; 163–178; 171–207; 324–342. Antenna 1250–1297 μm long, diameter of pedicel 185–188 (2) μm. AR 1.22–1.31.

*Thorax*. Antepronotals 6–10. Acrostichals 30–52, double staggered row which diverges posteriorly to join the dorsocentral row; dorsocentrals 24–38, biserial anteriorly and uniserial posteriorly; prealars 11–12 (2); supraalar 1 (1). Anapleural suture ratio 0.48–0.55. Scutellars 10–14. Scutal tubercle present.

**Figure 1.**
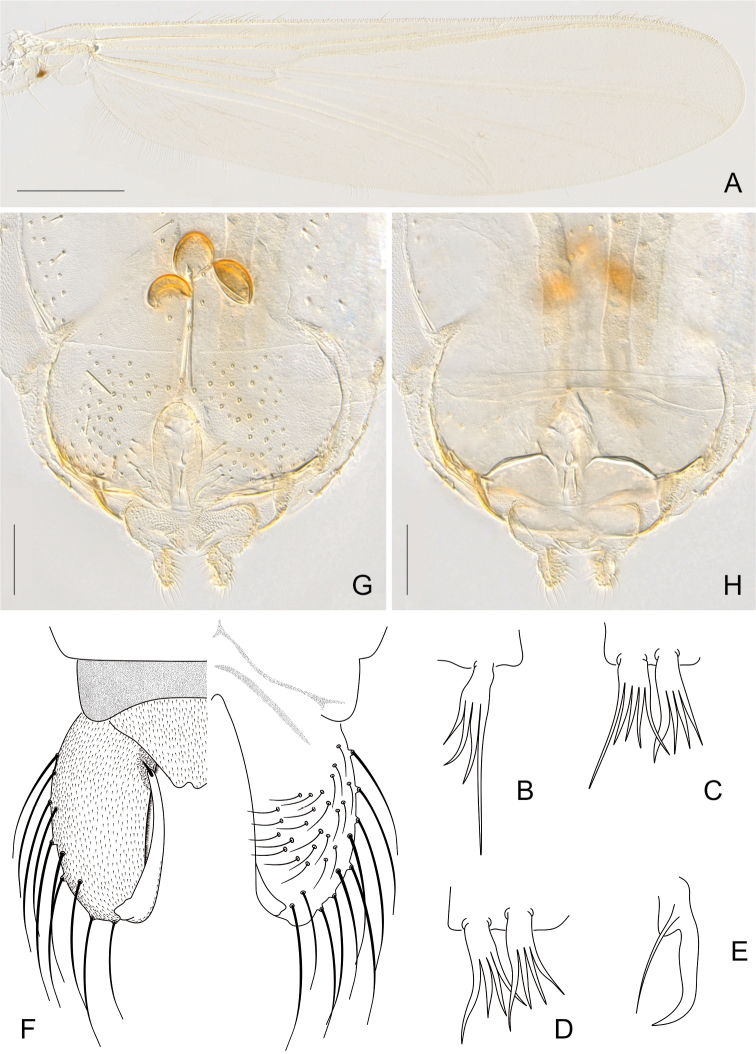
*Pentaneurellakatterjokki* Fittkau & Murray, adult male (**A–F**), adult female (**G–H**). **A** Wing **B** fore tibial spur **C** mid tibial spurs **D** hind tibial spurs **E** tarsal claw **F** hypopygium, left: ventral aspect, right: dorsal aspect **G** female genitalia, dorsal aspect **H** female genitalia, ventral aspect. Scale bars: 500 µm (**A**); 100 µm (**G, H**).

*Wing* (Fig. 1A). Width 0.85–0.86 (4) mm. Membrane densely covered with macrotrichia. Costa 2.9–3.1 (4) mm long without extension ending proximal to R_4+5_; MCu almost at FCu; R_2+3_ present, R_3_ not reaching costa. VR 0.91–1.05. WW 0.27–0.29. Brachiolum with 3 setae. Squama with 18–26 (2) setae. Anal lobe moderately developed.

*Legs* (Fig. 1B–E). Fore leg: width at apex of tibia 70 (2) μm, tibia with single, apical and pectinate spur 36–37 (2) μm long (Fig. 1B), with 4 (2) lateral teeth; ta_1-4_ without preapical pseudospurs. Mid leg: width at apex of tibia 64–72 μm, tibia with two apical spurs 22–27; 32–40 μm long (Fig. 1C), with 4–5 lateral teeth; ta_1-4_ with preapical pseudospurs. Hind leg: width at apex of tibia 62–72 μm, tibia with two apical spurs 22–29; 25–29 μm long (Fig. 1D), with 4 lateral teeth; comb not observed; ta_1-4_ without preapical pseudospurs. Claws slender, distally recurved and pointed and with large basal protuberance (Fig. 1E). Pulvilli absent. Lengths and proportion of leg segments as in Table 1.

*Hypopygium* (Fig. 1F). Tergite IX slightly concave posteriorly, without posterior setae. Membranous anal point broad, apically notched. Phallapodeme 106–130 (2) μm long. Sternapodeme with reduced anterior process. Gonocoxite cylindrical, 235–285 μm long, 103–131 μm wide. GcR 2.02–2.44. Gonostylus slender, 166–176 μm long, megaseta cochleariform, 14–17 μm long. HR 1.44–1.62. HV 3.04 (1).

**Table 1.** Lengths (in μm) and proportions of leg segments in *Pentaneurellakatterjokki* Fittkau & Murray, male (n = 2 or 3).

	fe	ti	ta_1_	ta_2_	ta_3_
p_1_	1385–1487	1433–1634	616–630	551–568	385–409
p_2_	1246–1503	1414–1757	935–1110	577–693	420–510
p_3_	1279–1304	1434–1519	1025–1163	684–732	471–507
	ta_4_	ta_5_	LR	BV	SV
p_1_	225–240	146–155	0.38–0.44	2.59–2.78	4.63–4.90
p_2_	241–284	154–182	0.63–0.75	2.34–2.77	2.22–3.12
p_3_	268–295	167–186	0.71–0.77	2.28–2.35	2.42–2.65

***Adult female* (n = 2, except where otherwise stated)**. *Size*. Total length 5.2 (1) mm. Wing length 3.3–3.4 mm. Total length/wing length 1.02–1.14. Wing length/profemur length 2.55–2.74.

*General coloration*. Head pale brown with darker occipital margin; pedicel and antenna brown; maxillary palp pale brown. Thorax pale brown. Wing membrane transparent without marks. Legs brown to pale brown. Abdominal tergites and genitalia pale brown.

*Head*. Temporal setae 22–24, irregularly uniserial. Eye ratio 1.12–1.33. Tentorium 186–287 μm long. Clypeus 157–176 μm long, 108–121 μm wide at largest part, bearing 25–28 setae. Cibarial pump 281–305 μm long. Lengths of palpomeres 1–5 (in μm): 52–57; 98–102; 180–181; 188–193; 333–334. Antenna 897–920 μm long, diameter of pedicel 98–100 μm. AR 0.36–0.39.

*Thorax*. Antepronotals 7. Acrostichals 44–48, double staggered row which diverges posteriorly to join the dorsocentral row of setae; dorsocentrals 36–48, biserial anteriorly and uniserial posteriorly; prealars 15–16; supraalars 2. Anapleural suture ratio 0.49 (1). Scutellars 8–10. Scutal tubercle present.

*Wing*. Width 1.00–1.10 mm. Costa 3.3–3.4 mm long. VR 0.96–0.98. WW 0.30–0.31. Brachiolum with 3 setae. Squama with 22–25 setae.

*Legs*. Fore leg: width at apex of tibia 71–74 μm, tibia with single, apical and pectinate spur 27–28 μm long, with 4 lateral teeth; ta_1-4_ without preapical pseudospurs. Mid leg: width at apex of tibia 54–68 μm, tibia with two apical spurs 27–28; 29–32 μm long, with 4–5 lateral teeth; ta_1-4_ with preapical pseudospurs. Hind leg: width at apex of tibia 65–67 μm, tibia with two apical spurs 30–35; 44–48 μm long, with 4 lateral teeth; comb not observed; ta_1-4_ without preapical pseudospurs. Claws slender, distally recurved and pointed and with large basal protuberance. Lengths and proportion of leg segments as in Table 2.

*Genitalia* (Fig. 1G–H). Gonapophysis VIII triangular, 84–85 μm long. Tergite IX without setae. Coxosternapodeme 142–159 μm long. Postgenital plate broadly rounded. Cerci oval-quadrate, 55–63 μm long, 24–31 μm wide; with 20 elongated setae. Labia with inconspicuous microtrichia. Notum 196–199 μm long. Seminal capsules oblong, 74–75 μm long, 55–69 μm wide, with conical shaped necks. Length ratio SCa/No 0.37–0.38.

**Table 2.** Lengths (in μm) and proportions of leg segments in *Pentaneurellakatterjokki* Fittkau & Murray, female (n = 2).

	fe	ti	ta_1_	ta_2_	ta_3_
p_1_	1235–1343	1539–1625	1149–1151	745–747	508–512
p_2_	1349–1414	1520–1621	822–1165	586–729	417–515
p_3_	1238–1448	1596–1879	837–1171	583–765	421–553
	ta_4_	ta_5_	LR	BV	SV
p_1_	290–306	175–196	0.71–0.75	2.28–2.34	2.41–2.58
p_2_	232–317	165–188	0.54–0.72	2.36–2.68	2.55–3.57
p_3_	258–286	158–182	0.52–0.62	2.40–2.74	2.66–3.64

***Pupa* (n = 2, except where otherwise stated).***Size*. Abdomen 3.5–4.3 mm long in male.

*General coloration*. Exuviae mostly pale brown without any distinctive patterns; thoracic horn brown.

**Figure 2.**
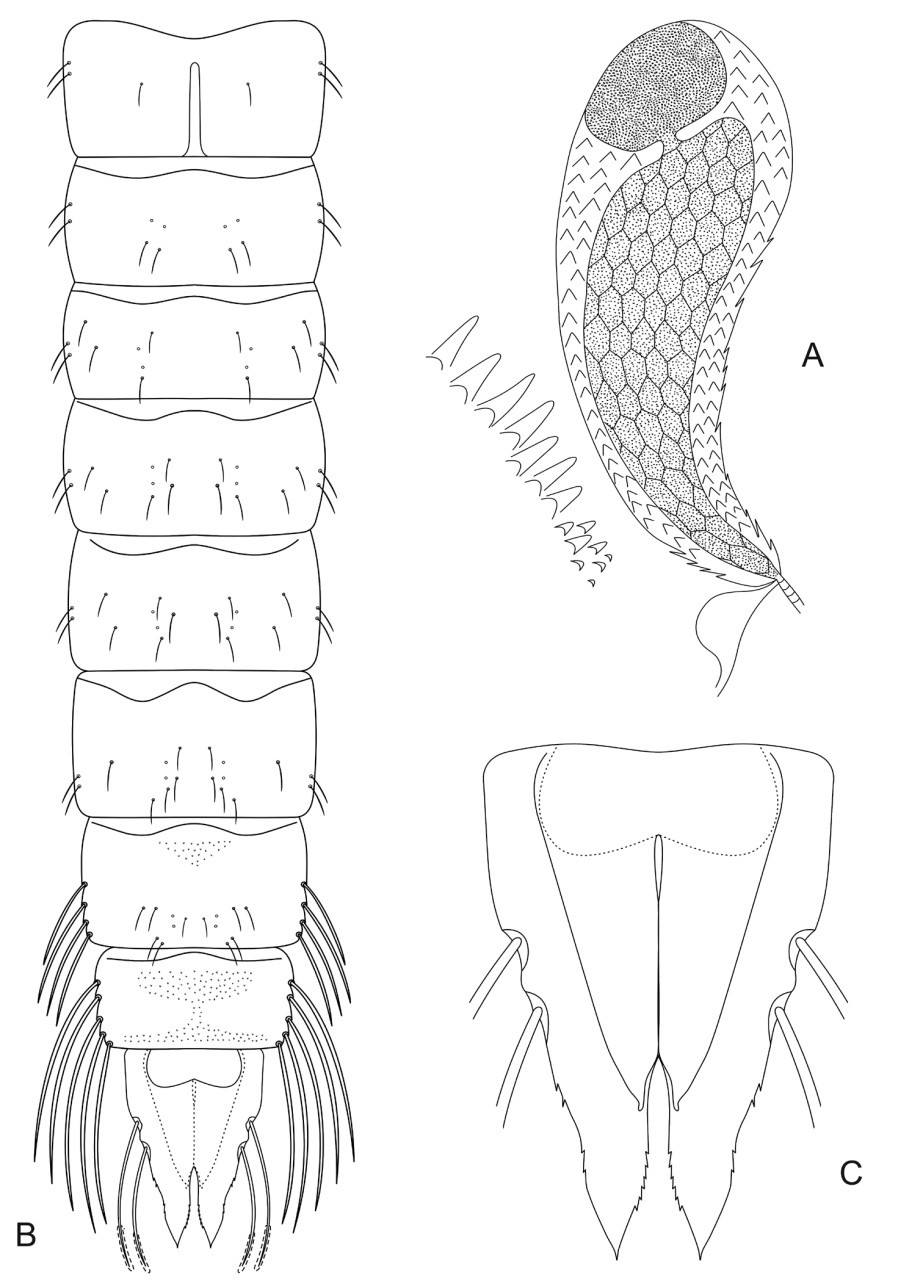
*Pentaneurellakatterjokki* Fittkau & Murray, pupa. **A** Thoracic horn with basal lobe and thoracic comb **B** abdominal segments with chaetotaxy, dorsal aspect **C** anal lobe and male genital sac, ventral aspect.

*Cephalothorax* (Fig. 2A). Wing sheath smooth, 1.5–1.6 mm long. Thoracic horn 322–336 μm long and 111–115 μm wide (Fig. 2A). THR 2.89–2.93. Respiratory atrium almost filling the lumen cavity, apically constricted into a narrow, short and straight neck, connected basally to a large rounded plastron plate. External membrane with spinules basally interconnected, forming scales. Basal lobe large apically round. Thoracic comb with 14 or 15 conical tubercles (Fig. 2A).

*Abdomen* (Fig. 2B, C). Tergite I with scar 217–238 μm long. Shagreen on tergites very sparse, spinules only present on the anterior median border of tergite VII, anterior and posterior borders of tergite VIII and sparsely on the anal lobe. Sternites I and VIII without shagreen, S II–VI with lateral longitudinal narrow bands or shagreen, S VII almost entirely covered with shagreen. Abdominal chaetotaxy as in figure 2B. Abdominal segment VII with 4 LS-setae. A VIII with 5 LS-setae. Anal lobe 516–533 μm long, 319–331 μm wide (Fig. 2C). ALR 1.60–1.62. Male genital sac not surpassing apex of anal lobe.

***4^th^ instar larva* (n = 2, except where otherwise stated).***General coloration*. Head golden yellow, postoccipital margin brown. Ligula pale yellow, with apex brown. Abdomen pale yellow. Procercus pale brown along anterior margin.

**Figure 3.**
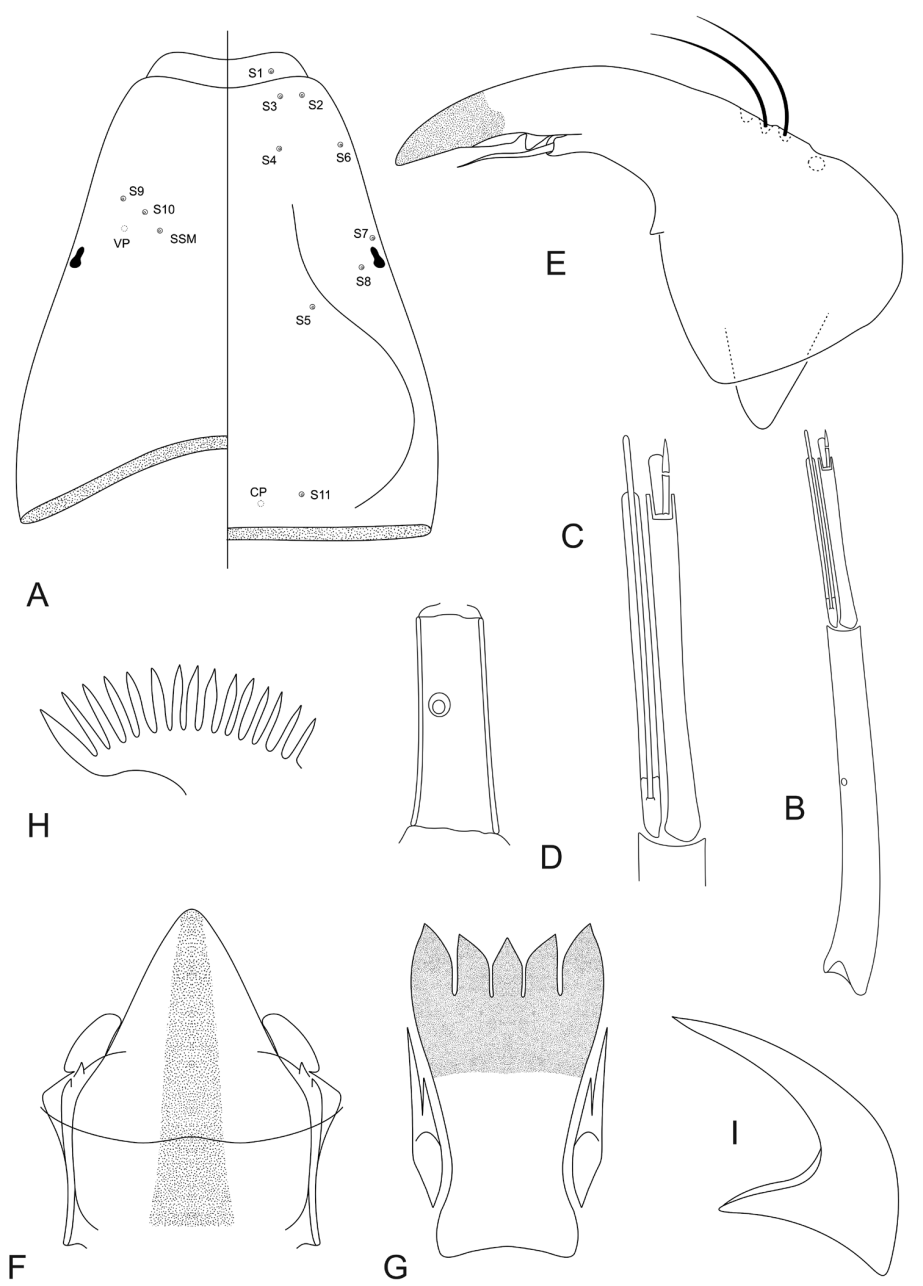
*Pentaneurellakatterjokki* Fittkau & Murray, larva. **A** Head with chaetotaxy. Left: ventral aspect, right: dorsal aspect **B** antenna **C** apex of antenna **D** maxillary palp **E** mandible **F** mentum and M-appendage **G** ligula and paraligula **H** pecten hypopharyngis **I** simple claw of posterior parapod.

*Head* (Fig. 3A). Length 808–873 μm, 495–518 μm wide; IC 0.59–0.61. Dorsally DP absent, S5 and S8 postero-mesal to S7. Ventrally S9, S10 and SSm forming a gently curved line (Fig. 3A).

*Antenna* (Fig. 3B–C). Length 374–383 μm, A_1_ 274–276 μm long, with ring organ located 0.44–0.54 from base, A_2_ 86–93 μm long. Peg sensilla large, firmly fused with margin of antennal segment 2, forming a fork-like process (Fig. 3C). AR 2.57–2.73. Blade 104–106 μm long.

*Maxilla* (Fig. 3D). Basal palp segment 59–60 μm long, 12–13 wide at middle, with ring organ located 0.40–0.68 from base. PR 4.44–4.45. APR 4.59–4.66.

*Mandible* (Fig. 3E). Length 95–115 μm. Sensillum campaniformium located 0.72 from apex. AMD 2.38– 2.89.

*Mentum and M appendage* (Fig. 3F). Dorsomentum sclerotised, without teeth. Labial vesicles oblong. Pseudoradula with fine granulation, not arranged in distinct longitudinal rows, 115 (1) μm long.

*Hypopharyngealcomplex* (Fig. 3G–H). Ligula with 5 teeth, 86–98 μm long, 44–52 μm wide at base; row of teeth slightly concave, middle and inner teeth subequal in size, outer slightly larger; inner teeth curved outward (Fig. 3G). IO 0.98–1.02, MO 0.98–1.00. Paraligula bifid, 36–47 μm long, inner tooth 29–36 μm long. Pecten hypopharyngis with 14–15 subequal teeth, corner tooth and middle teeth slightly broader than remainder (Fig. 3H).

*Body* (Fig. 3I). Without fringe of swim-setae. Procercus 153–182 μm long, 54–71 μm wide, with 6 anal setae 680–760 μm long. L/W 2.56–2.83. Anal tubules slender, 311–341 μm long. Posterior parapod 671–695 μm long. Claws simple (Fig. 3I), some with small spines on inner and/or outer margin.

#### Systematics.

In their comprehensive analyses of the Chironomidae subfamily Tanypodinae, Silva and Ekrem (2016) considered morphological characters across all life stages for all nine tribes within the subfamily, involving 54 genera and 115 species. In their study, Silva and Ekrem suggested that *Paramerina* Fittkau, *Reomyia* Roback and *Schineriella* Murray & Fittkau should be subgenera in *Zavrelimyia*. In addition, *Pentaneurella* was recovered as sister to *Trissopelopia* Kieffer in both analyses of equally weighted characters and by using implied weights. However, in an ongoing phylogenetic study of the subfamily Tanypodinae, which includes morphological evidence from modern and fossil chironomids (Silva and Baranov unpub. data), *Pentaneurella* turned out to be more closely related to the subgenera *Reomyia*, *Schineriella* and *Zavrelimyia*, within *Zavrelimyia* sensu Silva and Ekrem (2016), than to *Trissopelopia*, although with low support.

The male of *Pentaneurella* is morphologically similar to *Larsia* Fittkau, *Pentaneura* Philippi, *Trissopelopia*, and *Zavrelimyia* Fittkau. The bases of the lyrate tibial spurs are similar to the ones of *Larsia* and *Pentaneura*, and the absence of setae on tergite IX resembles *Trissopelopia* (Murray and Fittkau 1989). Nonetheless, *Pentaneurella* differs from *Larsia* in the presence of a distinctively notched, membranous anal point, while the presence of a distinct scutal tubercle separates adults of *Pentaneurella* from both *Trissopelopia* and *Pentaneura*. Moreover, *Pentaneurella* appears to be similar to *Zavrelimyia* sensu lato, only differing from the latter by having a scutal tubercle.

Regarding the immature stages, the pupa of *Pentaneurella* shows certain similarities to *Krenopelopia* Fittkau and *Monopelopia* Fittkau (Fittkau and Murray 1986). *Monopelopia* was recovered by Silva and Ekrem (2016) as sister to *Nilotanypus* Kieffer, and these two taxa as sister to Monopelopia (Cantopelopia) Roback. The presence of a basal lobe and thoracic comb, and anal macrosetae with adhesive sheaths, however, may be used to distinguish *Pentaneurella* from *Krenopelopia* and *Monopelopia* (Fittkau and Murray 1986). Larvae of *Pentaneurella* and *Krenopelopia* differ from *Pentaneura* and *Trissopelopia* by possessing a large peg sensilla which is firmly fused with the margin of antennal segment 2, forming a tuning-fork-like process. In both *Pentaneurella* and *Krenopelopia* the ligula has a lower middle tooth and inner teeth are curved outward. The absence of a dorsal pore, however, separates *Pentaneurella* from this genus (Cranston and Epler 2013). In addition, larvae of *Pentaneurella* appear to have cephalic setation and fork-like Lauterborn organs similar to those of *Zavrelimyia* sensu lato.

#### Remarks on distribution and ecology.

In the Palaearctic, the subfamily Tanypodinae is represented by 29 genera, of which *Anatopynia*, Johannsen, *Telmatopelopia* Fittkau and *Pentaneurella* currently are unique to the region. The latter is a relatively common genus of non-biting midges initially recorded from northern Scandinavia. Currently, the genus has been recorded in Finland (Paasivirta 2014), France (Brown et al. 2007, Moubayed-Breil et al. 2012), Germany (Stur and Wiedenbrug 2006), Norway (Fittkau and Murray 1983), Russia (Ashe and O’Connor 2009), Slovakia (Šporka 2003), Spain (Hjorth-Andersen 2002), Sweden (Fittkau and Murray 1983, Bylén and Ronny-Larsson 1994), Switzerland (Lods-Crozet 1998) and Turkey (Özkan 2006, Kazanci et al. 2008). Herein, we record *Pentaneurella* from Central Norway. Several specimens were collected in the Rondane National Park, located in typical high mountain area, with large plateaus and several lentic and lotic systems.

Little is known about the ecology of *Pentaneurella*. Immature stages seem to be cold stenothermic rheophiles and krenophiles. Larvae of *Pentaneurella* have been recorded inhabiting springs and spring-fed streams in Sweden and the Bavarian Alps as well as mountain streams in northern and Central Norway (Fittkau and Murray 1983, Stur and Wiedenbrug 2006, own data). Moubayed-Breil et al. (2012) found *Pentaneurella* in low and middle mountain streams located in the eastern Pyrenees and Corsica, while Bylén and Ronny-Larsson (1994) recorded larvae of *Pentaneurella* being parasitized by the microsporidium *Pernicivesiculagracilis* Bylén & Ronny-Larsson, in a sample of midge larvae collected from a small river in southern Sweden. Furthermore, larvae of *Pentaneurella* were also recorded from a sand bed stream from insular Turkey (Özkan 2006).

## Supplementary Material

XML Treatment for Pentaneurella katterjokki
